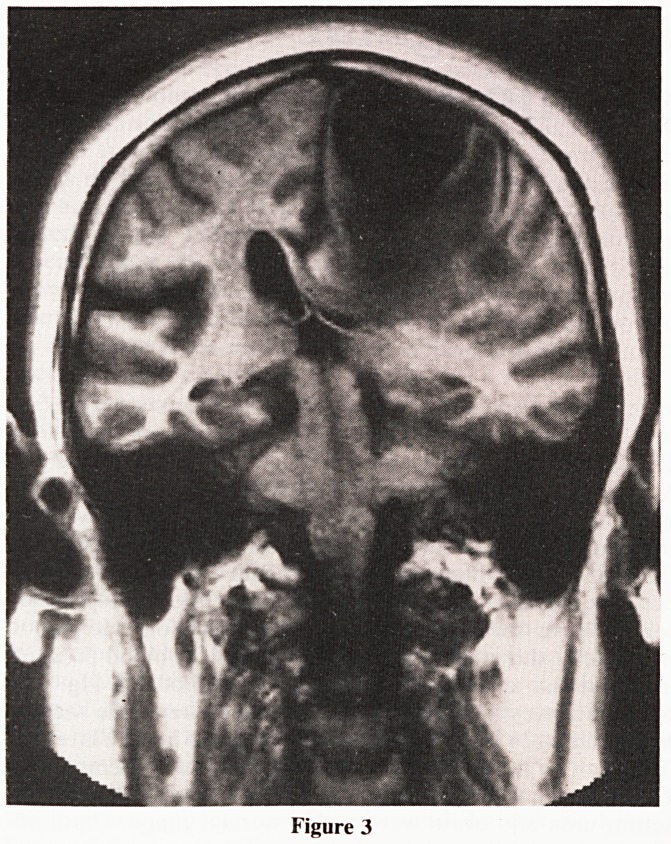# Imaging of Intracranial Tumours—Comparison of Computed Tomography and Magnetic Resonance Imaging

**Published:** 1990-12

**Authors:** M. Kuriakose

**Affiliations:** Medical Student, Bristol University


### Imaging of Intracranial Tumours—Comparison of Computed Tomography and Magnetic Resonance Imaging

**M. Kuriakose***
Medical Student, Bristol University

### INTRODUCTION

Magnetic resonance imaging (MRI) was first introduced by Paul Lauterbur in 1973, the same year that Hounsfield and Ambrose reported their initial experience with computed tomography (CT). While the subsequent development and acceptance of MRI lagged behind, the introduction of CT changed for all time the investigation of brain pathology. Recently there has been a dramatic improvement in MR imaging quality and it has achieved a capability for evaluating the normal and pathological states of the Central Nervous System (CNS) that not only rivals that of CT, but may even surpass it. The better clinical acceptance of MRI is because of its excellent contrast resolution, ability to perform multiplanar acquisition, and the lack of ionising radiation.

The advantage of MRI over CT are most obvious when imaging CNS. MRI is more sensitive than CT to changes in brain morphology and in turn more sensitive to the presence of tumours (Brant-Zawadski, 1988). This overview briefly compare CT and MR imaging of the intracranial tumours.

### PRINCIPLES OF CT AND MR IMAGING

CT scan images are produced by computerized reconstruction of a slice of tissues which has been analysed by a moving X-ray beam. The density of image depends on the X-ray attenuation properties of tissue, which in turn dependent on tissue atomic number and physical density. Air is shown as black and bone as white, with all intervening densities varying shades of grey.

MRI is similar to CT in that sectional images are produced, but the physical and biological principles are entirely different. MRI depends on the magnetic spin properties of nuclei (principally tissue Hydrogen) and the recovery of these nuclei after excitation with radio frequency (RF) electromagnetic waves. The nuclei excited by RF pulse, emit a radio-wave signal, the signal has a characteristic temporal profile. This depends on the number of hydrogen nuclei, their ability to exchange thermal energy in their molecular environment (T1 relaxation), the magnetic homogeneity of that environment (T2 relaxation), and the rate of motion of the hydrogen nuclei if any (flow effect). It is this multifactorial input into signal intensity that make MR imaging superior to CT in the depiction of tissue.

### GENERAL FEATURES OF INTRACRANIAL TUMOUR IMAGES

The morbidity and mortality potential of an intracranial neoplasm differ from those of tumours else where in the body. Local compression and invasion are as important to the prognosis as the histological features. Therefore the anatomical localization of a tumour is all-important in the clinical diagnosis.

Metastatic carcinoma accounts for nearly 15 to 30 percent of all intracranial neoplasms. Lung and breast carcinomas are the major sources of cerebral metastases. Primary brain tumours account for about 1 percent of all deaths and about 9 percent of all neoplasms (Schochet, *et al.*, 1979). Although ultimately classified according to the cell of origin, primary intracranial tumours can be broadly subdivided into two major groups; firstly Neuroectodermal tumours (Gliomas and Medulloblastomas) and secondly those tumours derived from other structures within the cranial cavity. These include meningiomas, schwannomas, pitutary adenomas, craniopharyngiomas, pineal tumours and haemangiomas. The commonest primary tumour is derived from glial cells and varies greatly in the degree of malignancy. According to the histologic types they are classified into astrocytomas, oligodendroglioma and ependymomas.

**Figure 1**

Evaluation of brain tumours on CT and MRI depend on their specific abnormal features: (Fig: 1)

1. Mass effect
2. Oedema
3. Abnormal contrast enhancement
4. Haemorrhage
5. Calcification
6. Associated abnormalities such as hydrocephalus and bone errosion
7. Tissue characteristics of Tumour (Orrison, 1989).

This paper was awarded the 1990 Annual Undergraduate First Prize by the Royal College of Radiologists.

Mass effect may cause obliteration of cortical sulci, ventricular displacement, midline shift and trans-tentorial or uncal herniation. As MRI is more sensitive to changes in brain morphology, mass effect is better depicted in this image. Spread of a lesion across the midline through the corpus callosum is well demonstrated by MR imaging compared with CT. This is a good clue to the primary origin of the lesion, as the oedema associated with a tumour generally does not spread into the corpus callosum (Brant-Zawadzki, 1988).

Oedema surrounding the lesion is a common characteristic of neoplastic and infective processes of CNS. Oedema is identifiable on CT scanning as areas of decreased attenuation related to normal brain structure. In MRI oedema appears as a decreased signal intensity on T1 weighted images and increased intensity on T2. With both CT and MR separation of the tumour of the surrounding oedema is difficult. The central foci of prolonged T1 relaxation of a tumour image corresponding more accurately to regions of contrast enhancement on CT, surrounded by larger areas of T2 prolongation, seen occassionally as low-attenuation regions on CT. The latter regions suggest the presence of peripheral oedema (Brant-Zawadzki, 1984).

Contrast enhancement is a characteristic feature of brain tumours. This has been attributed to leakage of the contrast agent into the extra vascular space through regions of disruption of the blood brain barrier (Sage, 1982). Recent histologic studies have shown that pathological neovascularity and endothelial proliferation also contribute to this phenomenon (Earnest, *et al.*, 1988). Iodine containing agents are used for contrast enhancement in CT imaging. Cerebral tumours show variable contrast enhancement in CT. These include 1. homogeneous enhancement 2. ring enhancement 3. non homogeneous enhancement and 4. no contrast enhancement. Gadolinium-labelled diethylene triamine pentaacetic acid (Gd-DTPA), a stable chelate complex of a rare-earth ion with strong para magnetic properties, has been shown to be a safe and effective contrast agent for MR imaging (Felix, *et al.*, 1985; Claussen, *et al.*, 1985).

Tissue characteristics of the tumours varies according to their cellular nature and architecture. In general MRI is more sensitive than CT in demonstrating intratumoural characteristics. Cystic changes can be observed in certain cerebral tumours, particularly in colloid cyst, glioblastoma, haemangioblastoma and pituitary adenomas. Calcification is another frequent feature of many intracranial tumours, especially astrocytoma oligodendroglioma and meningioma. CT readily shows calcification as a high dense area whereas it is difficult to identify with MRI. Typical feature of calcification is a decreased signal on both T1 and T2 weighted images. Central necrosis and haemorrhage are also seen in some tumours. Epidermoids, dermoids, teratomas and lipomas may contain fat density, which can be well depicted in both CT and MRI.

### SPECIFIC FEATURES OF INTRACRANIAL TUMOUR IMAGES

### 1. Metastatic tumours

Intracranial metastases can occur in cerebral substance, meninges, and bones. On CT they may present as single or multiple areas of hypodensity, isodensity, or hyperdensity, depending on the nature of the tumour. Almost all metastatic deposits show definite contrast enhancement; however the enhancement pattern may be variable. Hydrocephalus is a frequent abnormality in meningeal carcinomatosis (Enzmann, *et al.*, 1978). Post Gd DTPA infusion MR imaging has been suggested to improve lesion detection and aid in better stereotactic biopsy (Healy, *et al.*, 1987).

### 2. Astrocytoma

On CT scanning most low grade astrocytomas appear as areas of decreased attentuation prior to contrast enhancement with or without calcification. Surrounding oedema is usually minimal. The contrast enhancement pattern is suggested to be related to the grade of neoplasm with minimal enhancement in grade I and frequent enhamcement in grade IV (Thomson, 1976). Astrocytomas on MRI are in general decreased in signal intensity or isointense on T1 weighted images. However they are characteristically of marked increased sigal intensity on T2, with a surrounding area of oedema. Contrast enhancement is also a feature of MR imaging. Even though a clue to the degree of malignancy can be obtained by the level of contrast enhancement, accurate histologic assessment remains the only means of grading the tumour and determining the tumour margins (Earnest, *et al.*, 1988). (Fig: 2.)

**Figure 2**

### 3. Oligodendroglioma

CT scan of oligodendroglioma prior to contrast enhancement demonstrates an area of decreased density with minimal mass effect and surrounding oedema. Calcification is present in approximately 90 percent of cases. Irregular contrast enhacement particularly at the tumour margins is a typical feature of oligodendroglioma (Vonofakos, *et al.*, 1979). MRI demonstrates variable signal intensities. A heavily calcified lesion may show decreased signal on both T1 and T2 weighted images.

### 4. Meningioma

The characteristic finding of meningioma on CT scan include a hyperdense broad based mass adjacent to the dura with lytic or sclerotic adjacent bony changes, marked mass effect, surrounding oedema, calcification and intense contrast enhancement. Minimal calcification, variable enhancement, irregular margins and marked oedema suggest a more aggressive tumour (New, *et al.*, 1982). Meningiomas can be difficult to evaluate by MRI as signal intensity is variable. It may be identified as a relative area of decreased intensity within a surrounding oedema with increased signal on T2 weighted images. Post gadolinium T1 weighted MRI shows marked contrast enhancement. (Fig: 3.)

Figure 3

**5. Schwannoma**
Schwannoma of the acoustic nerve is a common tumour originating in the internal auditory canal. It produces expansion, erosion and flaring of medial internal auditory canal. There is usually abnormal contrast enhancement on CT and MRI. With the CT scanning, relatively invasive and potentially painful gas cisternography may be necessary to demonstrate an intracanalicular acoustic neuroma. However it can be demonstrated well on Gd-DTPA enhanced MR images (Daniels, *et al.*, 1987).

**6. Pituitary adenoma**
Unenhanced CT scan of pituitary adenoma features an enlarged pituitary fossa containing material of slightly higher density than brain. Contrast enhancement is almost always present and some show cystic changes or calcification. MRI is superior to CT in anatomic definition and tumour localization, and in the full visualization of the relationships with the adjacent blood vessels and optic chiasma (Bradshaw, *et al.*, 1988). Microadenomas (lesions less than 10 mm diameter) can be demonstrated well in post contrast enhanced MRI as a focal defect in an enhanced gland. (Pojunas, *et al.*, 1986).

**7. Craniopharingioma**
The CT characteristic of this tumour include calcification, cystic area and contrast enhancement (Naidich, *et al.*, 1976). MRI may be relatively ineffective in the demonstration of small areas of calcification typical in craniopharyngioma. Signal intensity may be variable on T1 weighted images and markedly increased on T2 weighted images.

**8. Pineal tumours**
These neoplasms usually present on CT prior to contrast enhancement as hypodense or isodense mass. It demonstrate intense homogeneous contrast enhancement (Zimmerman, *et al.*, 1980a). Signal intensity is variable on MRI and may be increased on T1.

**9. Choroid plexus papilloma**
Choroid plexus papilloma is identified on CT as an area of increased density within the involved ventricle. Following contrast infusion, it shows intense contrast enhancement on CT scan. An increase in the size of ventricle is a typical feature of this tumour (Zimmerman, *et al.*, 1980b).

**10. Lymphoma**
The CT findings of Lymphoma are variable. It includes isodense or hypodense lesions that show abnormal contrast enhancement. Oedema is a common feature. Both porimary and secondary lymphomas are usually adjacent to the ventricles or subarachnoid spaces and it may be difficult to detect on CT (Jack, *et al.*, 1985). Lymphoma has a variable appearance on MRI, with either increased or decreased intensity in both T1 and T2 images. In effect Lymphoma can mimic any lesion on MRI.

**11. Haemangioblastoma**
Haemangioblastoma occurs most frequently in the posterior fossa. CT and MRI demonstrate a cystic mass with vascular nodule on the wall that enhances intensely. It may also feature intense contrast enhancement without the cystic component (Seeger, *et al.*, 1881).

**12. Medulloblastoma**
It is an infratentorial neoplasm most commonly seen in children. On CT scanning prior to contrast enhancement it may present as a hyperdense lesion and may show variability in density following contrast infusion. MRI demonstrates a homogeneous decreased signal on T1 and increased signal on T2.

**13. Epidermoid, Dermoid, Teratoma and Lipoma**
These four less common intracranial neoplasms contain fat with or without calcification. In general they show variable signal intensity and no contrast enhancement. Extradural epidermoid tumours are associated with a well defined lytic skull defect with a sclerotic margin. Intradural epidermoid tumours are usually hypodense on CT scanning. Rupture of an epidermoid tumour into the ventricle may demonstrate a fat-CSF level on CT (Laster, *et al.*, 1977). Dermoid tumours show more calcification than epidermoid tumours because of the presence of dental elements. They are most common in the midline of the posterior fossa and skull base. Teratoma is seen mainly in the midline, with most occurring in the region of pineal body. They show mixed densities due to the presence of various elements in the tumour. Intracranial lipomas demonstrate low attenuation value on CT scanning. With T1 weighted MR image it shows increased signal intensity.

### CONCLUSION
The broadened sensitivity of MR to alteration of normal tissue microstructure produced by neoplasms should allow earlier and more thorough, if not more specific, diagnosis of tumours than with CT. Improved sensitivity in detecting pathological condition with MRI constitutes the ability to acquire multiplanar images, more thorough depiction of tumour extent and superior delineation of associated abnormalities such as hydrocephalus. The inability to detect calcification in certain instances of MR images is a major drawback. MRI is contraindicated in patients with metal objects like aneurysm clips and pacemaker. Because of the tunnel shaped design, MRI apparatus gives claustropohobia in some patients. Other technical problem includes prolonged time for the acquisition of images. At present, poor access is the main limiting factor in the use of MRI.

### ACKNOWLEDGEMENT
I thank Dr. Bradshaw, consultant neuro-radiologist of Frenchay hospital for his help in preparing this paper and Frenchay MRI Centre their assistance.

*Continued on page 115*

### REFERENCES

Imaging of Intracranial Tumours *continued from page 113*

### REFERENCES

1. BRADSHAW, *et al.*, (1988) Magnetic resonance imaging of the CNS. *Bristol Med. Chir. J.* **103(ii)** 13–18.
2. BRANT-ZAWADSKI, *et al.*, (1984) Primary intracranial tumour imaging: a comparison of MRI and CT. *Radiology* **150**, 473–440.
3. BRANT-ZAWADSKI (1988) MR Imaging of the Brain. *Radiology* **166**, 1–10.
4. CLAUSSEN, C. *et al.*, (1985) Application of contrast agents in CT and MRI: their potential in imaging of brain tumours. *Neuroradiology* **27**, 164–171.
5. DANIELS, D. L., *et al.*, (1987) MR detection of tumour in the internal auditory canal. *AJR* **148(6)**, 1219–1222.
6. EARNEST, F., *et al.*, (1988) Cerebral astrocytomas: Histopathologic correlation of MR and CT contrast enhancement with stereo tactic biopsy. *Radiology* **166**, 823–827.
7. ENZMANN, D. R., *et al.*, (1978) Computed tomography in leptomeningeal spread of tumour. *J. Comput. Asst. Tomogr.* **2**, 448–455.
8. FELIX, R., *et al.*, (1985) Brain tumours: MR imaging with gadolinium-DTPA. *Radiology* **156**, 681–688.
9. HEALY, M. E., *et al.*, (1987) Increased detection of intracrnial metastases with intravenous Gd-DTPA. *Radiology* **165**, 619–624.
10. JACK, C. R., *et al.*, (1985) Radiographic findings on 32 cases of primary CNS lymphoma. *AJNR* **6**, 899–904.
11. LASTER, D. W., *et al.*, (1977) Epidermoid tumours with intraventricular and subarachnoid fat: report of two cases. *AJR* **128**, 504–507.
12. NAIDICH, T. P., *et al.*, (1976) Evaluation of sellar and parasellar masses by CT. *Radiology* **120**, 91–99.
13. NEW, P. F. J., *et al.*, (1982) Malignant meningioma: CT and histologic criteria, including a new CT sign. *AJNR* **3**, 267.
14. ORRISON, W. W. (1989) "Introduction to neuroimaging" Little Brown and company—Boston/Toronto pp 135.
15. POJUNAS, K. W., *et al.*, (1986) MR imaging of prolactin secreting microadenomas. *AJNR* **7**, 209–213.
16. SAGE, M. R. (1982) Blood-brain barrier: phenomenon of increasing importance to the imaging clinician. AJR 138: 887–898.
17. SEEGER, J. F., *et al.*, (1981) Computed tomographic and angiographic evaluation of haemangioblastomas. *Radiology* **138**, 65.
18. SOCHOCHET and McCORMACK (1979) "Essential neuropathology"—Appleton-Century-Crofts/New York. pp 101.
19. THOMSON, J. L. J. (1976) Computerised axial tomography and the diagnosis of glioma: a study of 100 consecutive histologically proved cases. *Clin. Radiol.* **27**, 431–441.
20. VONOFAKOS, D., *et al.*, (1979) Oligodendrogliomas: CT patterns with emphasis on features indicating malignancy. *J. Comput. Asst. Tomogr.* **3**: 783–788.
21. ZIMMERMAN, R. A., *et al.*, (1980a) CT of pineal, parapineal and histologically related tumours. *Radiology* **137**, 669–677.
22. ZIMMERMAN, R. A., *et al.*, (1980b) Computed tomography of acute intratumoural haemorrhage. *Radiology* **135**, 355–359.

## Figures and Tables

**Figure 1 f1:**
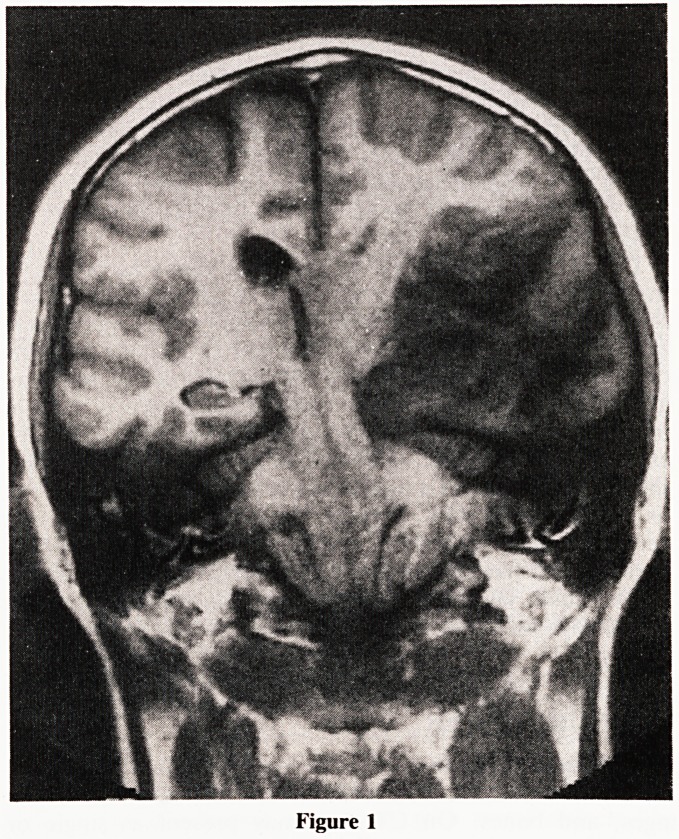


**Figure 2 f2:**
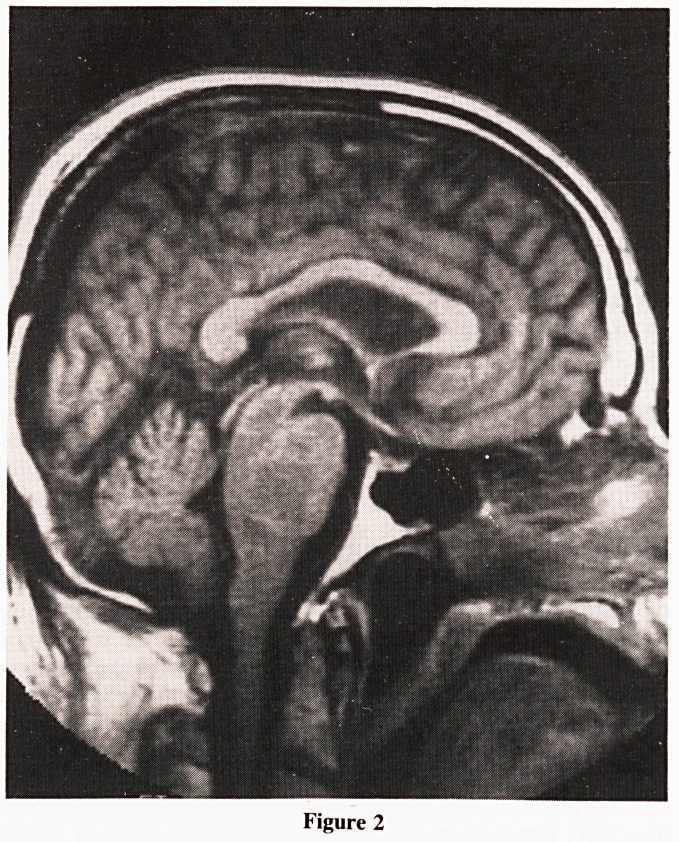


**Figure 3 f3:**